# Supplementary material for: Morphological and molecular characterization of an Elaeis oleifera (H.B.K) Cortes germplasm collection located in Ucayali, Peru
Source: PLoS One. 2021 May 6;16(5):e0250445. doi: 10.1371/journal.pone.0250445 (PMC8101761; doi:10.1371/journal.pone.0250445)
Supplement: S1 File — (DOC) [file pone.0250445.s002.doc]

**S1 File**

**Morphological and molecular characterization of an *Elaeis oleifera* (H.B.K) Cortes germplasm collection located in Ucayali, Peru.**

Alina Camacho-Villalobos^1^*, Fernando Serna^2†^, Jhofre Flores^3†^, Hector Flores^2^, Paulo Manrique^4^, Jorge Bendezu^5^*

^1^ Estación Experimental Agraria Pucallpa, Dirección de Desarrollo Tecnológico Agrario, Instituto Nacional de Innovación Agraria, Av. Centenario Km. 4 y 4.2, Coronel Portillo, Ucayali 25001, Perú.

^2^ Centro Experimental La Molina, Dirección de Recursos Genéticos y Biotecnología, Instituto Nacional de Innovación Agraria (INIA), Av. La Molina 1981, Lima, Lima 15024, Perú.

^3^ Facultad de Ciencias Agropecuarias, Universidad Nacional de Ucayali (UNU), Ucayali 25001, Perú

^4^ Independent research.

^5^ Estación Experimental Agraria Pucallpa, Dirección de Recursos Genéticos y Biotecnología, Instituto Nacional de Innovación Agraria (INIA), Av. Centenario Km. 4 y 4.2, Coronel Portillo, Ucayali 25001, Perú.

^*^Corresponding authors

^†^ Contributed equally

**E-mail:** [eduar156@yahoo.es](mailto:eduar156@yahoo.es); [acamacho@inia.gob.pe](mailto:acamacho@inia.gob.pe)

| **Trait** | **PC1** | **PC2** | **PC3** | **PC4** | **PC5** | **PC6** | **PC7** | **PC8** | **PC9** | **PC10** | **PC11** | **PC12** | **PC13** | **PC14** | **PC15** |
| --- | --- | --- | --- | --- | --- | --- | --- | --- | --- | --- | --- | --- | --- | --- | --- |
| **TH** | -0.12119 | **0.509121** | -0.08729 | **0.208583** | **0.294443** | 0.035143 | 0.112425 | -0.10624 | -0.13682 | **0.385805** | **-0.30357** | -0.18245 | **0.511876** | 0.067604 | 0.059369 |
| **TD** | -0.05834 | **0.506107** | 0.045719 | -0.01916 | -0.16874 | **0.2673** | -0.06231 | **0.408311** | 0.012086 | **-0.62082** | 0.159568 | -0.006 | **0.236786** | 0.0023 | 0.018205 |
| **CC** | **-**0.19956 | **0.549773** | -0.00435 | 0.142432 | 0.138602 | 0.013408 | -0.13111 | -0.17085 | -0.01855 | 0.03619 | -0.05437 | 0.18069 | **-0.72745** | -0.06202 | -0.03954 |
| **LL** | **-0.37285** | -0.04765 | **0.237613** | -0.03524 | -0.02074 | 0.197806 | **-0.22373** | 0.02878 | **0.496377** | **0.24348** | 0.021501 | **0.587192** | **0.227694** | 0.088125 | -0.0056 |
| **PL** | -0.15535 | -0.17805 | -0.01778 | **0.301255** | **0.458093** | **-0.2665** | **-0.64262** | -0.11331 | -0.00746 | **-0.30948** | 0.107936 | -0.13904 | 0.136762 | -0.0299 | -0.00137 |
| **LXL** | **-0.2268** | 0.105406 | **0.390666** | **-0.26371** | 0.094025 | 0.034336 | **0.211246** | **-0.50572** | -0.07215 | -0.0341 | **0.588803** | -0.19993 | 0.093721 | 0.02911 | -0.04721 |
| **LEL** | **-0.44214** | -0.08984 | 0.08089 | -0.02329 | -0.10907 | -0.00817 | -0.0168 | **0.274159** | **0.206252** | 0.085623 | -0.11168 | -0.54991 | -0.20763 | **0.541383** | -0.03152 |
| **LD** | -0.14384 | -0.03718 | -0.10497 | **0.5851** | **-0.43488** | -0.15294 | 0.054062 | -0.0478 | **-0.37074** | 0.070386 | **0.315853** | **0.242099** | 0.064498 | **0.319224** | 0.0023 |
| **LDW** | **-0.31839** | -0.04617 | 0.056072 | -0.06458 | 0.113199 | **-0.50354** | **0.462814** | -0.09622 | 0.020439 | -0.4332 | **-0.36373** | **0.264868** | 0.055829 | 0.075169 | -0.00725 |
| **FA** | **-0.41519** | -0.08567 | 0.081926 | **0.248063** | **-0.33938** | -0.00782 | 0.054557 | 0.012136 | 0.082691 | 0.04915 | -0.08096 | **-0.26213** | 0.021129 | **-0.73885** | 0.053038 |
| **BN** | -0.13238 | 0.048403 | **-0.48019** | **-0.26962** | **-0.35956** | 0.095299 | **-0.24782** | **-0.45819** | 0.023488 | -0.12649 | -0.19117 | -0.04121 | 0.123965 | 0.065148 | **-0.43901** |
| **ABW** | **-0.32111** | -0.15129 | **-0.27443** | -0.09224 | **0.368946** | 0.149486 | 0.180334 | **0.383953** | -0.2894 | 0.112691 | **0.222459** | 0.118431 | -0.02597 | -0.14419 | -0.51994 |
| **YP** | **-0.31263** | -0.08931 | **-0.48163** | **-0.26531** | 0.069267 | 0.145071 | -0.01019 | -0.03664 | -0.14532 | -0.01505 | 0.116546 | 0.068426 | -0.03306 | 0.005546 | **0.723811** |
| **FW** | 0.001587 | **-0.27269** | 0.03472 | **0.39293** | 0.189222 | **0.679618** | **0.243452** | **-0.27721** | 0.037007 | **-0.27707** | **-0.20523** | -0.02282 | -0.07369 | 0.100144 | 0.00198 |
| **FD** | 0.13851 | 0.104056 | **-0.46301** | **0.244382** | 0.107171 | -0.15614 | **0.291478** | -0.03145 | **0.659621** | -0.01181 | **0.357487** | -0.09551 | -0.00033 | -0.02078 | -0.0385 |
|  |  |  |  |  |  |  |  |  |  |  |  |  |  |  |  |
| **Standard**  **deviation** | 1.9753 | 1.4686 | 1.3808 | 1.20453 | 1.0927 | 0.946 | 0.88024 | 0.81298 | 0.75134 | 0.71506 | 0.66361 | 0.50714 | 0.42736 | 0.28294 | 0.15185 |
| **Proportion**  **of variance** | 0.2601 | 0.1438 | 0.1271 | 0.09673 | 0.0796 | 0.05966 | 0.05165 | 0.04406 | 0.03763 | 0.03409 | 0.02936 | 0.01715 | 0.01218 | 0.00534 | 0.00154 |
| **Cumulative**  **variance** | 0.2601 | 0.4039 | 0.531 | 0.62774 | 0.7074 | 0.76701 | 0.81866 | 0.86272 | 0.90036 | 0.93445 | 0.9638 | 0.98095 | 0.99313 | 0.99846 | 1 |

**S1 Table.** Principal component Analyses performed to Peruvian *E.oleifera* germplasm.

Value > 0.2 indicates the contribution of the trait to de components.

**Effect of null alleles**

**S2 Table.** Null allele frequencies for each microsatellite.

| **No** | **Microsatellite loci** | **Frequency** |
| --- | --- | --- |
| **1** | mEgCIR0353 | 0.0555556 |
| **2** | sM o00020 | 0.0000000 |
| **3** | mEgCIR3282 | 0.0138889 |
| **4** | mEgCIR0067 | 0.0416667 |
| **5** | mEgCIR3886 | 0.1805556* |
| **6** | mEgCIR0802 | 0.4861111* |
| **7** | mEgCIR0254 | 0.2777778* |
| **8** | mEgCIR0437 | 0.0000000 |
| **9** | mEgCIR3285 | 0.0000000 |
| **10** | mEgCIR0018 | 0.0555556 |
| **11** | sM o00129 | 0.2361111* |
| **12** | mEgCIR3546 | 0.3888889* |

Value > 0.1 indicates the presence null allele.

* Indicates the exclusion of this microsatellite to perform the effect of null alleles.

**S3 Table.** Impact of the null alleles in the genetic diversity.

| **Populations** | | **Hexp** | **H.sd** | **H.var.intra** | **Hexp.diff** | **p.value** |
| --- | --- | --- | --- | --- | --- | --- |
| Complete set (12 MS) | Set without null alleles (7 MS) | 0.6878644 | 0.1843939 | 0.0314097 | 0.0037411 | **0.3284399** |


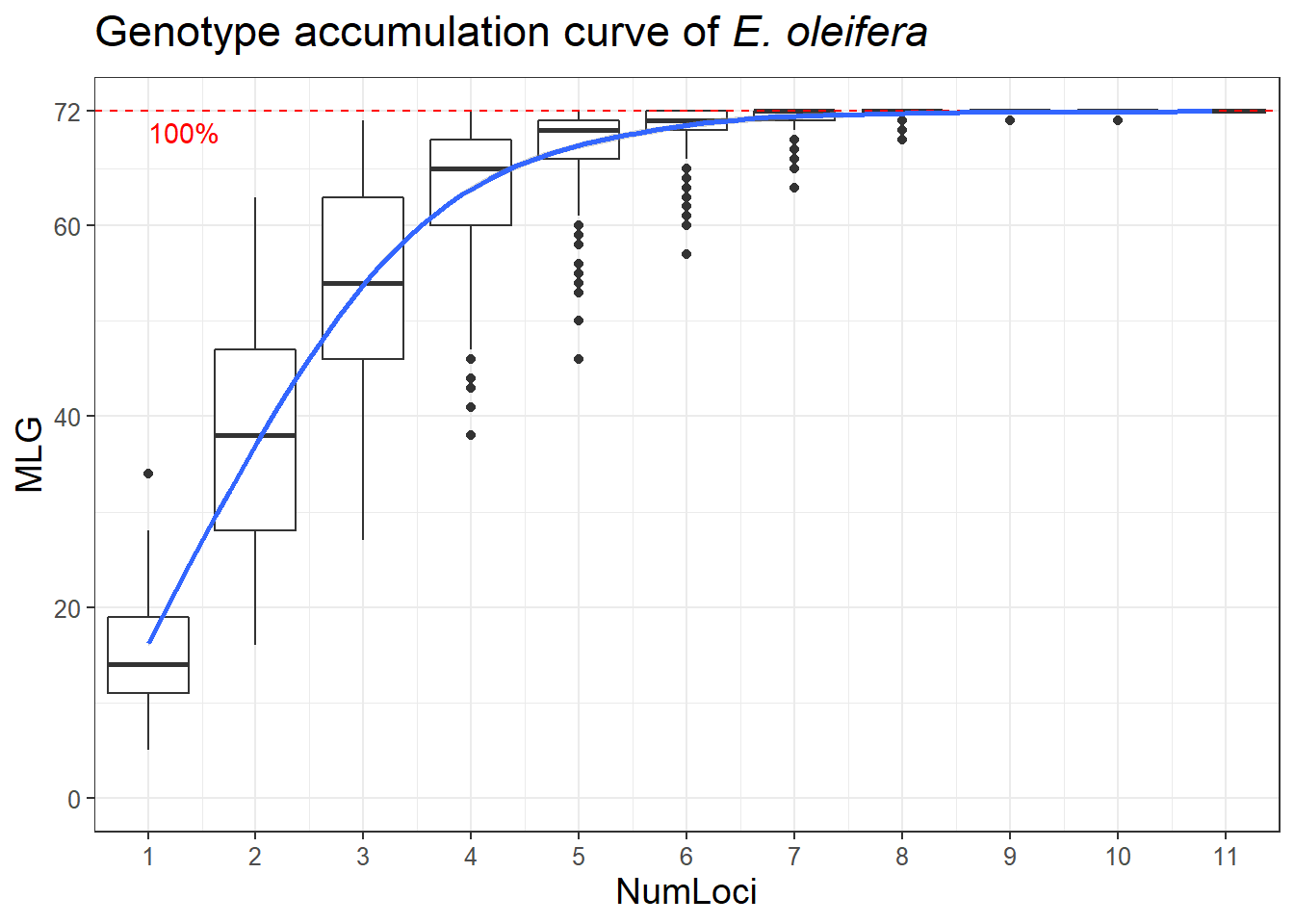


**S1 Fig.** Genotype accumulation curve. x-axis shows the number of loci used to calculate the number of multilocus genotypes. y-axis shows the number of multilocus genotypes founded per each iteration. Boxplots and dots represent the distribution of the 1,000 iterations run per each number of loci


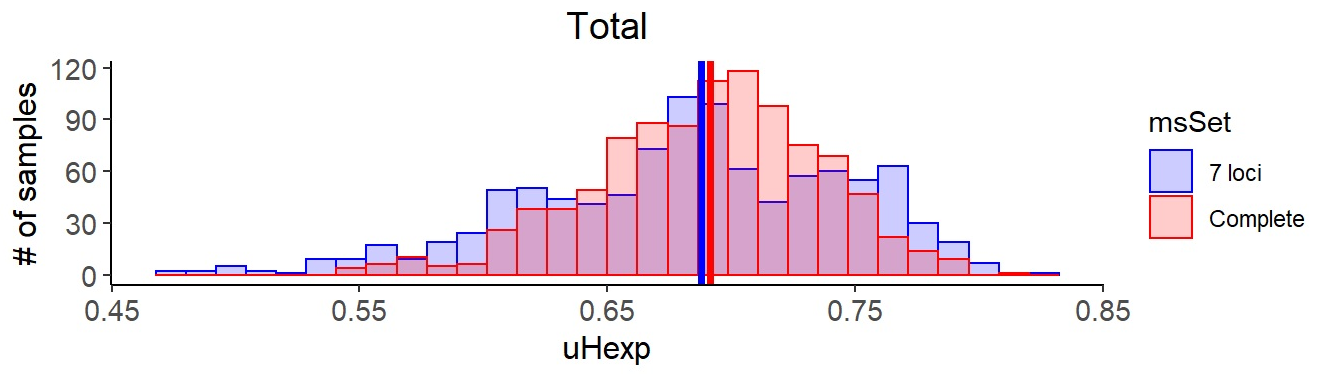


**S2 Fig.** Histogram distribution of the Hexp with the complete set of twelve microsatellites (Red) and seven microsatellites (Blue) with frequency ≤ 0.1 of null alleles. Difference between set of markers and statistical significances are in S4 Table.

**Additional references**

- Kamvar ZN, Brooks JC, Grunwald NJ. Novel R tools for analysis of genome-wide population genetic data with emphasis on clonality. Front Genet. 2015;6:208.
- Kamvar ZN, Tabima JF, Grunwald NJ. Poppr: an R package for genetic analysis of populations with clonal, partially clonal, and/or sexual reproduction. PeerJ. 2014;2:e281.
- Nei M, Roychoudhury AK. Sampling variances of heterozygosity and genetic distance. Genetics. 1974;76(2):379-90.
- Jombart T. adegenet: a R package for the multivariate analysis of genetic markers. Bioinformatics. 2008;24(11):1403-5.
- Jost L. GST and its relatives do not measure differentiation. Molecular ecology. 2008;17(18):4015-26.
- Winter DJ. MMOD: an R library for the calculation of population differentiation statistics. Molecular Ecology Resources. 2012;12(6):1158-60.
- Pritchard JK, Stephens M, Donnelly P. Inference of population structure using multilocus genotype data. Genetics. 2000;155(2):945-59.
- Evanno G, Regnaut S, Goudet J. Detecting the number of clusters of individuals using the software STRUCTURE: a simulation study. Mol Ecol. 2005;14(8):2611-20.
- Francis RM. POPHELPER: an R package and web app to analyse and visualize population structure. Molecular Ecology Resources. 2017;17(1):27-32.
- Jakobsson M, Rosenberg NA. CLUMPP: a cluster matching and permutation program for dealing with label switching and multimodality in analysis of population structure. Bioinformatics. 2007;23(14):1801-6.
