# Supplementary material for: Morphological and molecular characterization of an Elaeis oleifera (H.B.K) Cortes germplasm collection located in Ucayali, Peru
Source: PLoS One. 2021 May 6;16(5):e0250445. doi: 10.1371/journal.pone.0250445 (PMC8101761; doi:10.1371/journal.pone.0250445)
Supplement: Attachment — Submitted filename: Rebuttal Letter_Camacho-Villalobos A et al 2020fv.pdf [file pone.0250445.s003.pdf]

August 19th, 2020

Tzen-Yuh Chiang
Academic Editor
PLOS ONE

Subject: Submission of revised manuscript, PONE-D-20-17719

Dear Editor,

Thank you for your letter and the opportunity to revise our manuscript on **"Morphological and molecular characterization of an *Elaeis oleifera* (H.B.K. Cortes) germplasm collection located in Ucayali, Peru."** We highly appreciated the comments offered by the reviewers. We have carefully reviewed the editor's and reviewers' comments and have revised the manuscript accordingly.

Please, find below our point-by-point responses to reviewer's concerns. Changes to the manuscript are shown in track changes.

Additionally, we have updated the following information as it was requested:

1.- For the Funding Statement:

"This study was financially supported by Programa Nacional de Innovación Agraria (PNIA) of the Peruvian Government (http://www.pnia.gob.pe/) under the contract 042_PI. The funder provide support in the form of salaries for JF and HF and supplied materials for the study. Additionally, PM was supported by Independent Research in the form of salary. The funders had no role in study design, data collection and analysis, decision to publish, or preparation of the manuscript. The specific roles of these authors are articulated in the 'author contributions' section".

2.- For the Competing Interests Statement:

> *"PM is employed of Independent Research, and may own stock or hold stock options in that company. This does not alter the authors' adherence to PLOS ONE policies on sharing data and materials"*

We hope the revised version will better suit the PLOS ONE and are glad to consider further revisions. We thank you for your continued interest in our research.

We look forward to hearing from you at your earliest convenience.

Thank you for your consideration.

Yours sincerely,

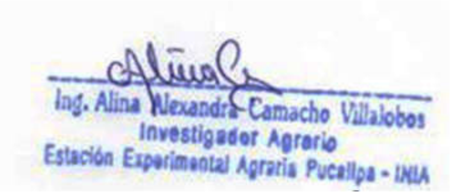

Estación Experimental Agraria Pucallpa

Instituto Nacional de Innovación Agraria (INIA)

Ucayali – Perú

Carretera Federico Basadre Km. 4.200
T: (51) 61-571831
E-mail: Pucallpa@inia.gob.pe
www.inia.gob.pe
www.minagri.gob.pe

Journal Requirements:

1. **Comment:** When submitting your revision, we need you to address these additional requirements. Please ensure that your manuscript meets PLOS ONE's style requirements, including those for file naming. The PLOS ONE style templates can be found at https://journals.plos.org/plosone/s/file?id=wjVg/PLOSOne_formatting_sample_main_body.pdf and https://journals.plos.org/plosone/s/file?id=ba62/PLOSOne_formatting_sample_title_authors_affiliations.pdf

   Response and change: We agree with the comment. We have revised diligently our manuscript and adapted it to PLOS ONE's style requirements.

2. **Comment:** Thank you for stating the following in the Competing Interests section: 'The authors have declared that no competing interests exist.' We note that one or more of the authors are employed by a commercial company: Independent research.

   2.1. Please provide an amended Funding Statement declaring this commercial affiliation, as well as a statement regarding the Role of Funders in your study. If the funding organization did not play a role in the study design, data collection and analysis, decision to publish, or preparation of the manuscript and only provided financial support in the form of authors' salaries and/or research materials, please review your statements relating to the author contributions, and ensure you have specifically and accurately indicated the role(s) that these authors had in your study. You can update author roles in the Author Contributions section of the online submission form. Please also include the following statement within your amended Funding Statement. "The funder provided support in the form of salaries for authors [insert relevant initials], but did not have any additional role in the study design, data collection and analysis, decision to publish, or preparation of the manuscript. The specific roles of these authors are articulated in the 'author contributions' section." If your commercial affiliation did play a role in your study, please state and explain this role within your updated Funding Statement.

Response and change: We thank the Editor's comment. We have updated our Funding Statement in the Financial Disclosure on the submission system.

Original text:

This work was financially supported by the Programa Nacional de Innovación Agraria (PNIA) of the Peruvian Government under the contract PI042-INIA-PNIA/UPMSI/IE.

Revised text:

"*This study was financially supported by Programa Nacional de Innovación Agraria (PNIA) of the Peruvian Government (http://www.pnia.gob.pe/) under the contract 042_PI. The funder provide support in the form of salaries for JF and HF and supplied materials for the study. Additionally, PM was supported by Independent Research in the form of salary. The funders had no role in study design, data collection and analysis, decision to publish, or preparation of the manuscript. The specific roles of these authors are articulated in the 'author contributions' section*".

2.2. Please also provide an updated Competing Interests Statement declaring this commercial affiliation along with any other relevant declarations relating to employment, consultancy, patents, products in development, or marketed products, etc. Within your Competing Interests Statement, please confirm that this commercial affiliation does not alter your adherence to all PLOS ONE policies on sharing data and materials by including the following statement: "This does not alter our adherence to PLOS ONE policies on sharing data and materials." (as detailed online in our guide for authors http://journals.plos.org/plosone/s/competing-interests). If this adherence statement is not accurate and there are restrictions on sharing of data and/or materials, please state

these. Please note that we cannot proceed with consideration of your article until this information has been declared.

Response and change: We thank the Editor's comment. We have updated the Competing Interests Statement declaring our commercial affiliation (Competing Interest section on the submission system).

Original text:

The authors have declared that no competing interests exist.

Revised text:

"*PM is employed of Independent Research, and may own stock or hold stock options in that company. This does not alter the authors' adherence to PLOS ONE policies on sharing data and materials*".

2.3. Please include both an updated Funding Statement and Competing Interests Statement in your cover letter. We will change the online submission form on your behalf. Please know it is PLOS ONE policy for corresponding authors to declare, on behalf of all authors, all potential competing interests for the purposes of transparency. PLOS defines a competing interest as anything that interferes with, or could reasonably be perceived as interfering with, the full and objective presentation, peer review, editorial decision-making, or publication of research or non-research articles submitted to one of the journals. Competing interests can be financial or non-financial, professional, or personal. Competing interests can arise in relationship to an organization or another person. Please follow this link to our website for more details on competing interests: http://journals.plos.org/plosone/s/competing-interests

Response and change: We thank the Editor's comment. We have included both an updated Funding Statement and a Competing Interests Statement in the cover letter.

2.4. Please include captions for your Supporting Information files at the end of your manuscript, and update any in-text citations to match accordingly. Please see our

Supporting Information guidelines for more information: http://journals.plos.org/plosone/s/supporting-information

Response and change: We agreed with the comment and we have included a list of supporting information and we updated the citations in the manuscript.

i) Text added **(page 21; lines 573-574)**

Supporting information captions

S1 Database Morphological - yield traits and SSRs_Camacho-Villalobos A *et al* 2020.

S2 File.

ii) Update citations to supporting information:

Material and Methods section; page 7; line 163: The complete collected phenotyping data is presented in **S1 file**.

Material and Methods section; page 8; line 195: The complete raw genetic data related to SSRs is showed in **S1 file**.

Results section; page 10; lines 229-231: The PCA analysis demonstrated that both the morphological and yield traits explained the variability in the oil palm individuals in this study (**S1 Table in S2 File**).

Results section; page 11; lines 267-273: The frequency of null alleles was estimated between 0.01 and 0.48 for nine microsatellites (**S2 Table in S2 File**). Then, five microsatellites that presented null alleles were excluded to perform a genotype accumulation curve and expected heterozygosity tests to identify the effect of null alleles in the subsequent analysis. The analysis showed that the use of at least seven microsatellites could identify the totality of the 72 multilocus genotypes identified (**S1 Fig in S2 File**), also the inclusion or exclusion of the null alleles in the analysis did not alter the final He ($p < 0.05$) in the Peruvian germplasm (**S2 Fig and S3 Table in S2 File**).

Discussion section; page 15; lines 363-367: Null alleles were previously reported in Peruvian samples with frequencies around 0.06 and 0.21 (1), and in this study were reported between 0.01 to 0.48, however, the presence of five microsatellites with more than 10% of null alleles (or missing information) do not alter the estimation of the genetic diversity, in terms of He, of the germplasm of 72 samples. (**S3 Table in S2 File**).

Discussion section; page 17; lines 420-422: … according to our analysis could be necessary at least 7 microsatellites in the Peruvian germplasm to carry up the complete analysis of genetic diversity (**S1 Fig and S2 Table in S2 File**), however many studies are necessary to corroborate these findings.

Response to Reviewer 1:

1. **Comment 1:** The study focuses on morphological, yield traits and genetic diversity of E.oleifera. However, the study on fruit related traits, which is an important commercial trait in oil palm is missing. Furthermore, according to descriptive statistics (Table 2), morphological traits has reduced variability than yield traits whereas yield traits has reduced variability than morphological traits in PCA analysis. There was a conflict between these two analyses. Is there any explanation for the observed results?

   Response and change: We thank the reviewer for the comment.

   We have focused the main idea of this article in the principal morphological and yield traits and some yield traits were related with fruit such as: i) the weight of fruits at harvest, ii) the number of fruits per palm during harvest, iii) weight in kg of 10 fruits, and iv) fruit diameter (**Material and Method section; Phenotyping).** These traits were described in American oil palm for genetic studies using molecular markers such as SSRs, in order to set up future studies in Peru that figure out the relationship between morphological and yield traits in this important crop using molecular markers. A similar study was recently published in 2019 by Osorio-Guarín J. et al. using SNPs https://doi.org/10.1186/s12870-019-2153-8. However, we have added some lines to refer this limitation in our study in the initial version of this manuscript (**Discussion section; page 13; lines 320-322.** Additionally, we count on with the fruit related traits and a

new manuscript is being prepared to present information related the fruit traits and quality in oil belong from this peruvian *E.oleifera* germplasm.

Regarding with the next sentence: *"Furthermore, according to descriptive statistics (Table 2), morphological traits has reduced variability than yield traits whereas yield traits has reduced variability than morphological traits in PCA analysis. There was a conflict between these two analyses. Is there any explanation for the observed results?"*. We have rephrased the whole paragraph in order to avoid the misinterpretation the results of variability. We emphasized in the results of descriptive statistics (Table 2) and PCA analysis, also we added a PCA results as a supplementary material to support our findings (**S1 Table in S2 File**).

Original text:

Descriptive statistics for the morphological and yield traits of the Peruvian *Elaeis oleifera* germplasm (n=72) are shown in Table 2. The morphological trait that exhibited the highest standard deviation (sd) was FA (35.48%, 13 073.0 – 127 688.0 cm2) compared with TH and CC, wich had standard deviations of 11.65% and 11.89%, respectively. Moreover, four of the five yield traits showed high variability (22.10 – 46.91%). These data show that the Peruvian *E.oleifera* germplasm has reduced variability compared with that of its yield traits.

To compare the variability among traits, a principal component analysis (PCA) was performed to identify the main traits that contributed to the dispersion of the values (Fig 1). Of the total variation in the Peruvian *E.oleifera* germplasm, was explained by two components: PC1 (26.01%), which was associated with four morphological traits (FA, LEL, LXL and LD) and two yield traits (YP and ABW) and PC2, which was primarily associated with two morphological traits (TH and CC) and explained the 40.39% of the variance in the Peruvian germplasm. The PCA analysis demonstrated that the morphological traits explained more of the variability in the oil palm individuals in this study than the yield traits.

Revised sentence: **Results section; page 9-10; lines 215-231**

Descriptive statistics for the morphological and yield traits of the Peruvian *Elaeis oleifera* germplasm (n=72) are shown in Table 2. The morphological trait that exhibited the highest

standard deviation (SD) was FA (SD: 20 894; min:13 073.0 – max: 127 688.0 cm^2; cv: 35.48%) and, one (YP) of the five yield traits showed high standard deviation (SD: 22.76; min: 0.6 – max: 22.60 mt; cv: 46.91%). These data show that both morphological and yield traits presented variability in the Peruvian germplasm.

A principal component analysis (PCA) was performed to identify the main traits that contributed to the dispersion of the values (Fig 1). Of the total variation in the Peruvian *E.oleifera* germplasm, was explained by two components: PC1 (26.01%), which was associated with five morphological traits (FA, LEL, LL, LXL, and LDW) and two yield traits (YP and ABW) and PC2, which was primarily associated with three morphological traits (TH, TD, and CC) and one yield trait (FW) explained the 40.39% of the variance in the Peruvian germplasm. The PCA analysis demonstrated that both the morphological and yield traits explained the variability in the oil palm individuals in this study (S1 Table in S2 File).

2. **Comment 2**. The author described the importance of relationship between the morphological traits and genetic diversity. According to their results, groups 1 and 3 were more closely related in morphological and yield traits whereas groups 1 and 2 were more closely related in genetic diversity analysis (number of alleles). Would appreciate more explanation for these results.

   Response and change: We appreciate the revisor´s comment. Regarding the genetic differences between groups 1, 2 and 3 (number of alleles), we had tested that difference to He and A between the groups using statistic parameter and we had not found any evidence to the differentiation between groups and we reported it in the first version of this manuscript **Discussion section; page 16; lines 390-394**. However, we have considered to include some lines to explain the lack of differences due to the low number of individuals for each group. And, we would like to emphasize in the use of neutral microsatellites to identified a correct set of diversity genetic parameters. **Discussion section; page 17; lines 403-405.**

3. **Comment 3**. The abstract can be made more reflective of the total paper. The authors need to highlight what are altogether the novel results of their study.

   Response and change: We thank the reviewer for the comment. We proceed to improve the abstract in order to highlight our novel results and its impact in the Peruvian industry. **Abstract section; pages 2-3; lines 46-71.**

4. **Comment 4**. Although previously published methodologies were referred, the methods and the number of replicates for measurement of phenotypic data in materials and methods section should be briefly described.

   Response and change: We thank the reviewer for the comment. we would like to rephrase a paragraph related to "Phenotyping" and add more information about the methods and the number of replicates that we employed in the study.

   Original text:
   All experimental data obtained were related to the morphological and yield characteristics of an American palm (*Elaeis oleifera* H.B.K. Cortes) plantation that was laid out using a traditional pattern, a quincunx, with plant and row spacing of 9 x 9 m. Nine morphological characters were quantified: Trunk Diameter (TD, trunk circumference at the midsection), Trunk Height (HT, distance between the lowest green leaves and the fruit), Cup Coverage (CC, Leaf number), Leaf Length (LL, the rachis length of leaf number 17), Petiole Length (PL), Leaf Dry Weight (LDW, mean dry weight per leaf multiplied by the number of leaves produced), Foliar Area (FA, mean area per leaf multiplied by the number of leaves per palm), Leaflet length (LL, length of the largest leaflet), Leafleat diameter (LD) and Leaflet per Leaf (LXL, number of leaflet per leaf). The five yield characters were: Bunch Weight (ABW, the weight of fruits at harvest), Bunch Number (BN, the number of fruits per palm during harvest), the 10-weight fruits (FW, weight in kg of 10 fruits), fruit diameter (FD) and Yield per Palm (YP, kg of fruit per palm per year). Each measure was made using previously published methodologies (2, 20, 21).

   Revised sentence: **Material and Methods section; page 6-7; lines 134-163**

   All experimental data obtained were related to the morphological and yield characteristics of an American palm (Elaeis oleifera H.B.K. Cortes) plantation that was laid out using a traditional pattern, a quincunx, with plant and row spacing of 9 x 9 m. Nine morphological characters were quantified: Trunk Diameter (TD was taken from the trunk circumference at the midsection with a tape from 10 cm below the crown height), Trunk Height (HT was estimated from the distance between the lowest green leaves and the fruit), Cup Coverage ((CC) The leaves were counted one by one following the phyllotaxis of the trunk), Leaf Length ((LL) the leaf located in the

middle third was taken, similar to the position of leaf number 17 in *E. guineensis*, once it was cut, the total length of petiole and length of the rachis were added to estimate the Leaf Length)), Petiole Length (PL was measured from petiole separated of the rachis in leaf number 17), Leaf Dry Weight (The dry weight were determined by chopping the leaf number 17 finely, and drying to constant temperature at 100-105ºC, them, the dry weight was multiplied by the number of leaves produced to estimate LDW), Foliar Area (FA represents the mean area per leaf multiplied by the number of leaves per palm this measure was obtained by measuring two leaves per palm), Leaflet length (LL was determinates in the largest leaflet in the leaf number 17 by measuring from apex to the insertion base of the rachis leaf), Leaflet diameter (LD was estimated by bending the largest leaflet in the middle and then width measure was taken) and Leaflet per Leaf (LXL, the number of leaflet per leaf was estimated by counting the leaflets in the leaf number 17 including rudimentary leaflets). The five yield characters were: Average Bunch Weight (ABW was determined by weighing the bunch at harvest time) , Bunch Number (BN was represented by the number of bunch per palm collected during the first harvest), the 10-weight fruits (FW was estimated by weighing 10 fruits at random), fruit diameter (FD) and Yield per Palm (YP, this indicator was calculated form the total production in kg of fruit per palm per year). Each measure was made using previously published methodologies (2, 20, 21). And, the number of replications for each morphological trait were done every two weeks during the year of evaluation with the exception of the LL, PL, LDW, and LD traits that were done once due to the implication of the use of destructive steps. The complete collected phenotyping data is presented in S1 file.

5. **Comment 5**. In the results section (Page 8, Line #186-187), it is not clear whether yield traits are compared to morphological traits or not. Please re-write that part.

   Response and change: We thank the reviewer for her/his observation. We agree with the reviewer; however, we decided eliminate this sentence in order to make a response to **Reviewer 1, comment 1.**

6. **Comment 6.** Page 11, Line # 261, the word "wich" should be "which"..).

   Response and change: We thank the reviewer for the correction. We agree and we proceed to corrected it. This change is shown in yellow color. **Discussion section; page 13; line 307**

7. **Comment 7.** Page 581, Table 4, the word "morphologycal" should be "morphological".

   Response and change: We thank the reviewer for the correction. We agree and we proceed to corrected it. This change is shown in yellow color. **Table 4**

8. **Comment 8.** Page 262, Line 262-263, please clearly describe the sentences.

   Response and change: We thank the reviewer for the observation. Also, we considered to add a sentence to explain the differences between Colombian and Peruvian germplasm.

   Original text:

   The primary phenotypic differences within this Peruvian germplasm were based on two morphological traits, trunk height (TH) and cup coverage (CC), wich explained 40.4% of the total variation in contrast, the variation in an Elaeis oleifera Colombian germplasm was explained by for four traits (trunk height, leaf area, foliar area and leaf dry weight) (2)

   Revised sentence: **Discussion section; page 13; lines 305-310**

   The primary phenotypic differences within this Peruvian germplasm were based on three morphological traits, trunk height (TH), trunk diameter (TD) and cup coverage (CC), and one yield trait (FW, Fruits 10-weight)which explained 40.4% of the total variation in contrast, the variation in an E. oleifera Colombian germplasm was explained by four morphological traits (trunk height, leaf area, foliar area, and leaf dry weight) (2) however, the age of both plantations could explain this differences.

9. **Comment 9**. English is sometimes odd, please improve it.

   Response and change: We thank the reviewer for his/her comment. We considered improving the English employing a professional language service. We attached the certificate of this service.

Carretera Federico Basadre Km. 4.200
T: (51) 61-571831
E-mail: Pucallpa@inia.gob.pe
www.inia.gob.pe
www.minagri.gob.pe

EL PERÚ PRIMERO

Response to Reviewer 2:

1. **Comment 1.** The manuscript describes genetic diversity of Elaeis oleifera assembled from an area in Peru by means of 12 SSRs and selected morphological traits. The manuscript was written in readable English language but with minor spelling mistakes and some grammatical errors. These errors are however correctable. I have specific concerns as below. The phenotypic data was collected at very young age, when the palms are 4 years old, as described by authors in method section. It should be cautioned that analysis and interpretation of such data can be erroneous and misleading. It is very important to note that For oil palm specifically, standard phenotypic data collection is carried out for at least 8 years, starting from palms at 2 or 3 years old. Measuring vegetative traits such as height, frond length is carried out once on palms at 8th year old. I am doubtful on the results presented especially if it is going to be used for the purposes of selection and breeding.

   Response and change: We thank the reviewer for his/her comment. Regarding to the English language we answered on the same way that the **reviewer 1, comment 9.**

   Regarding with the next sentence: *"The phenotypic data was collected at very young age, when the palms are 4 years old, as described by authors in method section. It should be cautioned that analysis and interpretation of such data can be erroneous and misleading. It is very important to note that For oil palm specifically, standard phenotypic data collection is carried out for at least 8 years, starting from palms at 2 or 3 years old. Measuring vegetative traits such as height, frond length is carried out once on palms at 8th year old. I am doubtful on the results presented especially if it is going to be used for the purposes of selection and breeding"*.

We agree with the reviewer and we have done some changes in the message of our manuscript in order to provide a realistic application of our results.

i) We clarified that the term "four-year-old" of the germplasms is since of transplanting event. **Material and methods section; page 6; lines 129-130**.

ii) The objective of our study was rephrased as follow:

Original Text

In that context, the aim of this study was to characterize the E. oleifera (H.B.K) Cortes germplasm of a Peruvian collection using morphological traits and SSR markers to further national breeding programs for the oil palm.

Revised sentence: **Introduction section; page 5; lines 118-121**

In that context, the aim of this study was to characterize the *E. oleifera* (H.B.K) Cortes germplasm of a Peruvian collection using morphological traits and SSR markers to contributed with important data for setting up of further studies that will be related to the development of national breeding programs for the oil palm.

iii) We eliminated a sentence and added a new paragraph to highlight the principal limitation of our study related to the age of the germplasm.

Eliminated Text: **Discussion section; page 17; lines 429-431**

This information must guide us in the decision-making process when planning breeding programs that are focused on crosses to obtain OxG interspecific hybrids (E. oleifera x E. guineensis) with interesting yield traits.

Added text: **Discussion section; page 18; lines 435-438**

However, this information should be taken carefully due the age of the germplasm (four-year-old) and further studies are necessary to complement it, by including new data trait (morphological and yield) corresponding to the next four years in according to other previous studies (1,20).

iv) The final paragraph of our study was rephrased as follow:

Original Text

Finally, the information presented here demonstrates that the 72 accessions of the Peruvian E. oleifera located in Ucayali region of Peru represent a useful genetic resource to promote the use of the genetic material and to design new national breeding strategies and conservation programs for this important crop in the tropical regions of Peru and South America.

Revised sentence: **Discussion section; page 18; lines 431-435**

Finally, this novel information presented here demonstrates that the 72 accessions of the Peruvian *E. oleifera* located in Ucayali region of Peru represent a useful genetic resource to promote the use of the genetic material and to promote and design further investigations to establish national breeding strategies and conservation programs for this important crop in the tropical regions of Peru and South America.

v)Additional revisions were made by the authors in the discussion section to make clear the scope of our results. **Discussion section; page 12; lines 293-295 and pages 15-16; lines 375-377.**

2. **Comment 2.** The challenge of using SSRs for genetic analysis is the occurrence of null alleles. Null alleles introduce error in assessment of genetic diversity, parentage analysis etc. The authors can carry out relevant analysis to identify null alleles, exclude them from dataset prior to genetic diversity analysis.

Response and change: We thank the reviewer for his/her comment. We agree with the comment and we tested the impact of null alleles in our analysis. Our additional analysis is described in the material and methods section and the supplementary data section, we found that five SSRs have null alleles and we excluded them and performed comparative tests between all complete set (12 SSRs) and 7 SSRs (without 5 SSRs), and our results showed that there are no significant differences. At the light of these results we decided to include this new information throughout the manuscript as follows:

i) We added a new paragraph in Material and methods section (**Materials and methods section; page 9; lines 207-211).**

Additionally, to test the effect of null alleles on the power of discrimination genotypes was addressed by the construction of a genotype accumulation curve with 1,000 iterations with replacement of samples, while the effect on the expected heterozygosity was addressed by a Monte Carlo test with 1,000 loci resampling considering the exclusion of loci with more than 10% of null alleles. All these procedures were tested using the R v3.42 software.

ii) We added a new paragraph in Results section (**Results section; page 11; lines 267-273).**

The frequency of null alleles was estimated between 0.01 and 0.48 for nine microsatellites (S1 Table in S2 File). Then, five microsatellites that presented null alleles were excluded to perform a genotype accumulation curve and expected heterozygosity tests to identify the effect of null alleles in the subsequent analysis. The analysis showed that the use of at least seven microsatellites could identify the totality of the 72 multilocus genotypes identified (S1 Fig in S2 File), also the inclusion or exclusion of the null alleles in the analysis not alter the final He ($p < 0.05$) in the Peruvian germplasm (S2 Fig and S3 Table in S2 File).

iii) We added a new paragraph in Discussion section (**Discussion section; page 15; lines 363-370).**

Null alleles were previously reported in Peruvian samples with frequencies around 0.06 and 0.21 (1), and in this study were reported between 0.01 to 0.48, however, the presence of five microsatellites with more than 10% of null alleles (or missing information) do not alter the estimation of the genetic diversity, in terms of He, of the germplasm of 72 samples. (S3 Table in S2 File). These alleles are produced by putative mutations that occur at the microsatellite binding sites that could prevent primer annealing and in consequence, the lack of amplification of the PCR products (8) therefore further primer development could be necessary for the local or regional *E. oleifera* germplasm.

iv) We considered to add a Supporting Information file to support our additional results related to null alleles (S2 table, S3 table, S1 Fig and S2 Fig) in according to reviewer 2, comment 2.

3. **Comment 3.** The number of SSRs applied is limited. The trend of publication nowadays applied hundreds or even thousands of markers for diversity analysis. I encourages authors to add more markers to achieve reasoanably acceptable statistics to support any conclusions made from the analysis.

Response and change: We really appreciate the reviewer for his/her recommendation. In order to achieve an evidence about the utility of our data, we performed an analysis by the construction of a genotype accumulation curve with 1,000 iterations with replacement of samples employed (n=72). Our results show us that the use at least 7 SSRs could show us the correct identification of 72 multilocus genotypes. Also, we identified two recent studies carried out in central (Ithnin M et al 2017 DOI 10.1186/s12863-017-0505-7) and south America (Arias D et al 2015 DOI 10.1007/s11295-015-0946-y) where were employed a small SSRs set (<20). In our study we employed twelve microsatellites and we could identify the 72 multilocus genotypes in the Peruvian *E. oleifera* germplasm according to our additional and exhaustive analysis.

Additionally, the large set of SSRs is necessary to the identification of QTLs associated with any special trait in oil palm and segregation studies for example Jeennor S and Volkaert S 2014 (DOI 10.1007/s11295-013-0655-3) reported the use of 83 SSR and 101 SNPs, and Osorio-Guarín J *et al* 2019 (doi.org/10.1186/s12870-019-2153-8) reported the use of 3776 SNPs without SSRs to identified QTLs in *E. oleifera.* For that in special to *E.oleifera* diversity genetic the use of SSRs at least in South America will tend to be constant, however, the use of new markers such as SNPs will be restricted to QTL identification according to recent reports (Osorio-Guarín J *et al* 2019).

At the light of these results we decided to include this new information throughout the manuscript as follows:

i) We added a new paragraph in Results section (**Results section; page 11; lines 267-273).**

The frequency of null alleles was estimated between 0.01 and 0.48 for nine microsatellites (S2 Table in S2 File). Then, five microsatellites that presented null alleles were excluded to perform a genotype accumulation curve and expected heterozygosity tests to identify the effect of null alleles in the subsequent analysis. The analysis showed that the use of at least seven microsatellites could identify the totality of the 72 multilocus genotypes identified (S1 Fig in S2 File), also the inclusion or exclusion of the null alleles in the analysis not alter the final He ($p < 0.05$) in the Peruvian germplasm (S2 Fig and S3 Table in S2 File).

ii) We added lines to a paragraph in Discussion section (**Discussion section; page 17; lines 417-422).**

Moreover, the limited economical resources in many laboratories in South America could interrupt the adoption of these molecular tools. Recently, there are studies in *E.oleifera* that show that useful information is achieved when is employed a limited set of microsatellites (<20 SSRs) (1,8) and according to our analysis could be necessary at least 7 microsatellites in the Peruvian germplasm to carry up the complete analysis of genetic diversity (S1 Fig and S3 Table in S2 File), however many studies are necessary to corroborate these findings.

iii) We considered to add a Supporting Information file (**S1 Fig. in S2 file**) to support our additional results related to null alleles in according to reviewer 2, comment 3.

Carretera Federico Basadre Km. 4.200
T: (51) 61-571831
E-mail: Pucallpa@inia.gob.pe
www.inia.gob.pe
www.minagri.gob.pe

iv) We would considerate to share the all database in order to allow the discussion between distinct groups about the data generated in this study. We added **S1 file** in supporting information section.

Carretera Federico Basadre Km. 4.200
T: (51) 61-571831
E-mail: Pucallpa@inia.gob.pe
www.inia.gob.pe
www.minagri.gob.pe

Language Editing Services

Registered Office:
Elsevier Ltd
The Boulevard, Langford Lane,
Kidlington, OX5 1GB, UK.
Registration No. 331566771

To whom it may concern

The paper "Morphological and molecular characterization of an Elaeis oleifera (H.B.K. Cortes) germplasm collection located in Ucayali, Peru" by Camacho-Villalobos, Alina was edited by Elsevier Language Editing Services.

Kind regards,

Elsevier Webshop Support

(This is a computer generated advice and does not require any signature)
